# Robotic‐assisted total knee arthroplasty is not associated with improved accuracy in implant position and alignment compared to conventional instrumentation in the execution of a preoperative digital plan

**DOI:** 10.1002/jeo2.12019

**Published:** 2024-04-02

**Authors:** Christian Nogalo, Luca Farinelli, Amit Meena, Fabrizio di Maria, Elisabeth Abermann, Christian Fink

**Affiliations:** ^1^ Gelenkpunkt—Sports and Joint Surgery FIFA Medical Centre of Excellence Innsbruck Austria; ^2^ Research Unit for Orthopaedic Sports Medicine and Injury Prevention (OSMI) UMIT TIROL—Private University For Health Sciences and Health Technology Hall in Tirol Austria; ^3^ Department of Clinical and Molecular Sciences Clinical Orthopaedics Ancona Italy; ^4^ Division of Orthopedics Shalby Hospital Jaipur India; ^5^ Department of General Surgery and Medical Surgical Specialties, Section of Orthopaedics and Traumatology, University Hospital Policlinico “Rodolico‐San Marco” University of Catania Catania Italy

**Keywords:** component positioning, conventional TKA, preoperative digital planning, robotic TKA, short‐term outcome

## Abstract

**Purpose:**

The primary objective of the present study was to evaluate if robotic‐assisted total knee arthroplasty (RO‐TKA) results in improved accuracy compared to conventional TKA (CO‐TKA) with respect to alignment and component positioning executing a preoperative digital plan. The secondary objective was to compare patient‐reported outcome measures (PROMs) between the two groups at 6 months of follow‐up (FU).

**Methods:**

Patients who underwent primary TKA using the concept of constitutional alignment were identified from the database. Each patient underwent preoperative digital planning as well as postoperative evaluation of the preoperative plan (alignment and component position) using mediCAD® software (Hectec GmbH). Two groups were formed: (i) The RO‐TKA group (*n* = 30) consisted of patients who underwent TKA with a robotic surgical system (ROSA®, Zimmer Biomet) and (ii) the CO‐TKA group (*n* = 67) consisted of patients who underwent TKA with conventional instrumentation. To assess accuracy, all qualitative variables were analysed using the *χ* ^2^ test. Tegner activity scale, Oxford Knee Score and visual analogue scale were assessed preop and at 6‐month FU. To assess differences between the two groups, a 2 × 2 repeated measures analysis of variance was performed.

**Results:**

There was no significant (*p* > 0.05) difference in the accuracy of alignment as well as tibial and femoral component position between the two groups. At the 6‐month FU, there was no significant (*p* > 0.05) difference in PROMs between the two groups.

**Conclusion:**

While robotic TKA may have some potential advantages, no significant difference was found between robotic and conventional TKA with respect to limb alignment, clinical outcomes and component positioning.

**Level of Evidence:**

Level III.

**Abbreviations**
ANOVA: analysis of variance
CO‐TKA: conventional total knee arthroplasty
FU: follow‐up
HKA: hip knee ankle
ICC: intraclass correlation coefficient
LDFA: lateral distal femoral angle
MPTA: medial proximal tibial angle
OA: osteoarthritis
OKS: Oxford Knee Score
PROMS: patient‐reported outcome measures
RO‐TKA: robotic total knee arthroplasty
SD: standard deviation
TKA: total knee arthroplasty
VAS: visual analogue scale

## INTRODUCTION

Total knee arthroplasty (TKA) is the gold standard treatment for end‐stage osteoarthritis (OA) [37]. The number of annual procedures is expected to increase in the future due to an increasing population and longer life expectancy [11, 30]. Although TKA aims to reduce pain and restore knee function, a significant number of patients are dissatisfied with residual symptoms after surgery [5, 7, 15]. One reason for the inferior clinical results of TKA may be altered joint kinematics.

Historically, mechanical alignment was used in TKA to ensure satisfactory implant survival rather than to restore native knee function [34]. However, mechanical alignment may not be a valuable choice for the full range of prearthritic knee anatomy [2, 3, 13] and may alter postoperative gait patterns. In young active patients suffering from knee arthritis, more normal knee kinematics seems essential. Therefore, recently a ‘personalised’ arthroplasty has been suggested to better restore knee function after knee arthroplasty [2, 10, 14, 21]. Many new alignment concepts have been popularised over the past years, and more detailed preoperative planning has become an important tool for most of these techniques [6, 12, 20, 21]. Conventional instrumentation for TKA has been traditionally designed for mechanical alignment and has hardly been adapted to facilitate the intraoperative execution of these new alignment concepts. Therefore, the use of navigation and especially robotics has been gaining more and more interest. Computer navigation and robotic arm (robotic‐assisted total knee arthroplasty [RO‐TKA]) increase surgical precision [19], but some complications and disadvantages are associated with robotic systems (i.e., pin‐hole fractures, longer operative time, higher cost) [24]. However, continuous efforts are being made to improve these complications.

Therefore, the primary objective of the present study was to evaluate if RO‐TKA results in improved accuracy compared to conventional TKA (CO‐TKA) with respect to component positioning and alignment executing a preoperative digital plan of constitutional alignment. In addition, operating times and intraoperative complications were also analysed. The secondary objective was to compare patient‐reported outcome measures (PROMs) of the RO‐ and CO‐TKA groups at the 6‐month follow‐up.

## METHODS

### Study design and patients

Data were collected prospectively for patients who underwent primary TKA using the concept of constitutional alignment from June 2021 to April 2022 [6]. The inclusion criteria were unilateral knee OA, age 50–80 years, completion of preoperative and postoperative radiographic evaluation (bilateral long‐leg weight‐bearing hip‐to‐ankle (HKA) anterior to posterior and a standard lateral radiograph pre‐ and postoperative), completion of preoperative planning with the concept of constitutional alignment using mediCAD® software version 5.98 (Hectec GmbH) [13]. A total of 97 patients fulfilled the inclusion criteria. Based on the patient database, two groups were formed post‐hoc: the RO‐TKA group (*n* = 30, 12 female, 18 male) consisted of patients who underwent TKA with a single robotic surgical system (ROSA® knee system, Zimmer Biomet). The CO‐TKA group (*n* = 67, 32 female, 35 male) consisted of patients who underwent TKA with conventional instrumentation. The study was conducted at Gelenkpunkt–Sports and Joint Surgery, FIFA Medical Centre of Excellence, Innsbruck and approved by the Ethics Committee of the Medical University of Innsbruck (No. 1149/2023).

### Preoperative planning

Each patient in both groups underwent preoperative digital planning as well as postoperative evaluation of the preoperative plan using mediCAD® software, version 5.98 (Hectec GmbH). To quantify the differences between the preoperative deformity and the postoperative overall alignment, the HKA angle, the mechanical lateral distal femoral angle (LDFA) and the medial proximal tibial angle (MPTA) were analysed [25]. Digital planning was performed by a single knee fellowship‐trained orthopaedic surgeon (E. A.).

Preoperative planning was performed according to the principles of constitutional alignment by Bonnin et al. The goal was to restore the native patient‐specific, prearthritic limb (HKA) and joint alignment [6]. However, ‘safe zones’ for TKA alignment were used. LDFA and MPTA were set between ±5° and 90°. HKA was limited within 3° of neutral.

### Surgical procedure and rehabilitation

All surgeries were performed by a single experienced knee fellowship‐trained orthopaedic surgeon (C. F.). A medial parapatellar approach and a cemented, cruciate‐retaining Persona (Zimmer Biomet Inc.) total knee prosthesis were implanted in either group. No patella resurfacing was done. A tourniquet was used in all patients and tourniquet time as well as total surgical time was recorded. During the cement setting the leg was positioned in extension and closure was not started until the cement was fully hardened.

All patients were mobilised on the day of surgery and underwent a standardised postoperative rehabilitation programme. Patients were advised to use crutches with weight bearing as tolerated for about 4 weeks. Patients were evaluated preoperative and at 6 months postoperative for patient‐reported outcomes, activity level and knee pain. The Tegner Activity Scale [31], Oxford Knee Score (OKS) [36] and visual analogue scale (VAS) [8] were used.

### Statistical analysis

Data were analysed descriptively using numbers and percentages to present categorical data. Mean and standard deviation were used to summarise numerical variables. Based on the central limit theorem and after a graphical consideration of the distribution of the data, the data were considered to be normally distributed, and statistical tests were chosen for metric variables.

To assess accuracy, all qualitative variables were analysed using the *χ* ^2^ test with Yates correction when the sample size was less than 5. The significance level was set at *p* = 0.05. The differences in preoperative planning and postoperative outcome of each surgical method are presented as frequencies in the respective subgroups of deviations. The subgroups were divided into <1°, 2–3° and >3°. To assess differences between the two groups (RO‐TKA vs. CO‐TKA) with respect to PROMS, a 2 × 2 repeated measures analysis of variance was performed to detect differences over time (baseline and 6 months) and between groups. To evaluate the agreement between the intraclass correlation coefficient (ICC) of the mean HKA, MPTA and LDFA, a 95% confidence interval was used. An independent samples *t* test was used to determine if there was a difference between the surgery and tourniquet time in the groups. Statistical analysis was performed using the SPSS software package (IBM SPSS Statistics Version 29).

## RESULTS

The present study included 30 and 67 patients in the RO‐TKA and CO‐TKA groups, respectively. Both groups did not differ significantly (*p* < 0.05) with respect to demographics (age, gender, body mass index), preoperative deformity (HKA, MPTA and LDFA) and PROMS. Descriptive information regarding the sociodemographic and baseline measures of HKA, MPTA and LDFA are summarised in Table 1. The ICC correlation with its 95% confidence interval of HKA, MPTA and LDFA, was 1.000 (1.000–1.000), 0.999 (0.998–1.000) and 0.998 (0.996–0.999), respectively.

**Table 1:** Mean values (±SD) of the confounders (age, sex and BMI) and the reference values for the leg axis HKA; MPTA and LDFA.

	Group ‘robotic’ (*n* = 30)		Group ‘conventional’ (*n* = 67)		
	Mean ± SD	Min–Max	Mean ± SD	Min–Max	*p* Value
Age (years)	68.40 ± 7.15	45–80	68.25 ± 9.20	41–82	0.153
Gender (M/F)	18/12	/	35/32	/	0.478
BMI (kg/m^2^)	23.89 ± 3.75	17–31	27.71 ± 4.77	18–42	0.440
HKA (°)	0.15 ± 2.47	−4.8 to 4.4	−1.26 ± 2.27	−7.2 to 3.5	0.386
MPTA (°)	88.32 ± 1.90	85.2–91.6	87.78 ± 1.80	83.2–91.6	0.072
LDFA (°)	88.31 ± 1.66	85.5–90.9	89.08 ± 1.74	85.7–92.4	0.123

Abbreviations: BMI, body mass index; HKA, hip knee ankle; LDFA, lateral distal femoral angle; MPTA, medial proximal tibial angle.

**Figure 1:**
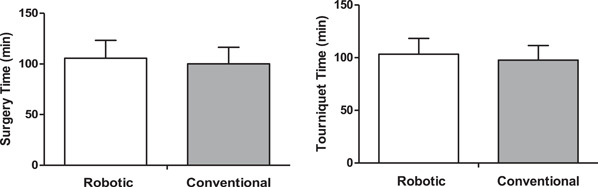
Mean values (±SD) surgery time and tourniquet time between robotic total knee arthroplasty and conventional total knee arthroplasty techniques.

There was no significant difference (*p* > 0.05) between RO‐TKA (105.63 min ± 17.64 min/103.37 min ± 14.97 min) and CO‐TKA (99.72 min ± 16.04 min/97.70 min ± 13.85 min) in terms of operating (*p* = 0.107) and tourniquet time (*p* = 0.073); (Figure 1). No intra or postoperative complications have been reported in either group.

### HKA

In 12 patients (40.0%) of the RO‐TKA group postoperative HKA differed less than 1° from the preoperative plan, in eight patients (26.7%) it differed between 1° and 2° and in six patients (20.0%) between 2° and 3°. In the CO‐TKA group, the results for HKA difference were 16 (23.9%), 24 (35,8%) and 13 (19.4%), respectively. Overall, 26 patients (86.7%) of the RO‐TKA group and 53 (79.1%) of the CO‐TKA group were within 3° of variation from the preoperative plan with respect to HKA. Four patients (13.3%) of the RO‐TKA group and 14 (20.9%) of the CO‐TKA group deviated greater than 3° from the preoperative plan. There was no significant (*p* > 0.05) difference between the two groups (Table 2).

**Table 2:** The differences in accuracy between HKA, MPTA and LDFA between a robotic and conventional group from preoperative digital planning.

	Robotic group	Conventional group
	No. patient (%), *N* = 30	No. patient (%), *N* = 67
HKA angle		
ΔHKA ≤ 1°	12 (40.0%)	16 (23.9%)
ΔHKA (>1° and ≤2°)	8 (26.7%)	24 (35.8%)
ΔHKA (>2° and ≤3°)	6 (20.0%)	13 (19.4%)
ΔHKA > 3°	4 (13.3%)	14 (20.9%)
*p* Value	0.386	
MPTA		
ΔMPTA ≤ 1°	15 (50.0%)	20 (29.9%)
ΔMPTA (>1° and ≤2°)	11 (36.7%)	24 (35.8%)
ΔMPTA (>2° and ≤3°)	4 (13.3%)	14 (20.9%)
ΔMPTA > 3°	0 (0.0%)	9 (13.4%)
*p* Value	0.072	
LDFA		
ΔLDFA ≤ 1°	12 (40.0%)	34 (50.7%)
ΔLDFA (>1° and ≤2°)	11 (36.7%)	17 (25.4%)
ΔLDFA (>2° and ≤3°)	7 (23.3%)	9 (13.4%)
ΔLDFA > 3°	0 (0.0%)	7 (10.4%)
*p* Value	0.123	

Abbreviations: Δ, the difference between planning and postoperative results; BMI, body mass index; HKA, hip knee ankle; LDFA, lateral distal femoral angle; MPTA, medial proximal tibial angle.

### Tibial component

For the RO‐TKA group positioning of the tibial component measured with MPTA differed less than 1° in 15 patients (50.0%), between 1° and 2° in 11 patients (36.7%) and between 2° and 3° in four patients (13.3%) from the preoperative plan. In the CO‐TKA group, the results were 20 (29.9%), 24 (35.8%) and 14 (20.9%), respectively. While all patients in the RO‐TKA group were within 3° of variation to the preoperative plan, in nine patients (13.4%) of the CO‐TKA group the difference was greater than 3° for tibial component placement. However, this difference did not reach significance (*p* > 0.05) (Table 2).

### Femoral component

Femoral component position evaluated by LDFA differed less than 1° in 12 patients (40.0%), between 1° and 2° in 11 patients (36.7%) and between 2° to 3° in seven patients (23.3%) in the RO‐TKA group. The results for the CO‐TKA group were 34 (50.7%), 17 (25.4%) and nine (13.4%), respectively. In the RO‐TKA no difference greater than 3° to the preoperative plan could be found, whereas this difference was present in seven (10.0%) of patients of the CO‐TKA group. The differences between the groups were not significant (*p* > 0.05) (Table 2).

### PROMs

**Figure 2:**
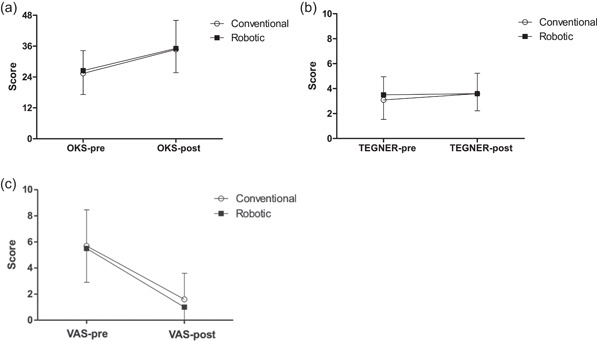
Mean values (±SD) of Oxford Knee Score (OKS) (a), Tegner activity level (b) and visual analogue scale (VAS) (c) obtained before (pre) and after (post) conventional (open circles; *n* = 67) or robotic (closed squares: *n* = 30) knee surgical replacement. (▪ robotic; ◦ conventional).

Significant improvements over time were found for OKS (*p* = 0.001) and VAS (*p* = 0.001) in both groups but not for the Tegner Activity Level score (*p* = 0.095). No significant group differences were detected for OKS (*p* = 0.763), VAS (*p* = 0.596) and Tegner activity level (*p* = 0.137). Details are summarised in Figure 2.

## DISCUSSION

The main finding of the present study was that the use of a robotic surgical system (ROSA® knee system; Zimmer Biomet) was not associated with improved accuracy in executing a preoperative digital plan of constitutional knee alignment compared to an experienced knee surgeon using conventional instrumentation. RO‐TKA needs more tourniquet and surgical times compared to CO‐TKA, but these differences were not statistical difference. No intra‐ or postoperative complications were found in either group. No differences were reported in PROMS between the two groups at short‐term follow‐up. Hence, no significant differences in terms of postoperative alignment (HKA) and component positioning have been detected between robotic and conventional TKA replacement. There was a tendency for a reduction of component positioning errors greater than 3° for the RO‐TKA group (0% for the RO‐TKA group compared to 13.4% (tibia) and 10.4% (femur) for the CO‐TKA group), but this difference did not reach significance.

Implementation of computer technology in TKA is a constantly evolving process to optimise surgical outcomes, patient safety, efficiency and cost‐effectiveness [1, 9, 16, 17, 18, 19, 27, 32, 35]. Augmented reality, patient‐specific instrumentation and robotic systems should assist the surgeon in the exact placement of appropriate cutting guides. Several studies reported that the use of ROSA® knee system (Zimmer Biomet) makes the surgeon more accurate and reproducible in bone resections compared to conventional instrumentation [26, 28].

Parratte et al. reported differences below 1° between planned and measured axes in 30 cadaver knees [26]. Seidenstein et al. compared 20 conventional to 14 robotically assisted TKA using the ROSA system. HKA was within 3° in all robotic‐assisted TKAs in comparison to only 75.0% of the conventional TKA. Moreover, considering 2° as the target, 92.9% of ROSA TKA would be in the target compared to 60.0% in the control group [29]. However, a common limit of their studies was the use of cadaveric specimens, which typically have less OA and deformities than clinical cases. In a recent paper, Schrednitzki et al. described a mean difference between the planned and measured axis of 1.01° ± 0.08°. There were no outliers regarding the ±3° target and almost no outliers regarding the 2° target [29]. The current study showed that four patients (13.3%) of the RO‐TKA group and 14 (20.9%) of the CO‐TKA group deviated greater than 3° from the preoperatively planned HKA. Moreover, as regards MPTA angle, all patients in the RO‐TKA group were within 3° of variation to the preoperative plan while nine patients (13.4%) of the CO‐TKA group were outliers (>3°) for tibial component placement. Similar to the conventional LDFA angle, no outliers (>3° from planning) have been found in the RO‐TKA group, while seven (10.0%) of patients were outliers from the conventional group.

A recent meta‐analysis reported that the robotic approach did not provide a relevant improvement compared to conventional manual TKA implanted in terms of clinical and radiological outcomes [4]. However, it needs to be highlighted that all the studies included in the metanalysis aimed at the mechanical alignment of TKA. On the other hand, a recent study by Turan et al. showed that the robotic‐assisted technique could perform restricted kinematic alignment more accurately, with significantly better radiographic outcomes in comparison to the manual technique. However, it is important to note that the Navio Surgical system (Smith & Nephew) has been used [33].

In our study, no differences in clinical outcome with respect to VAS, Oxford and Tegner Score could be found between the two groups. Conversely, Mulpur et al. reported significant differences in terms of clinical results between robotic and manual TKA in simultaneous knee replacement [23]. However, the differences were not clinically relevant. Mitchell et al. found significant early clinical benefits with robotic TKA, including lower opioid requirements, and shorter length of stay, when compared with conventional TKA [22]. However, a recent meta‐analysis reported no relevant difference in the clinical results, therefore a clear superiority of RO‐TKA over CO‐TKA could not be demonstrated [4].

The present study had few limitations. The total number of patients is relatively small. Due to the retrospective analysis of the study, the selection between CO‐TKA and RO‐TKA was not randomised. All plannings were performed by one experienced orthopaedic surgeon and all surgeries were performed by one experienced surgeon. This fact does eliminate confounding factors but results cannot be generalised. Finally, the length of follow‐up is short.

## CONCLUSION

The clinical benefits of robotic‐assisted TKA are still controversial. From the present study, no significant difference was found between robotic and conventional TKA with respect to limb alignment, clinical outcomes and component positioning. Increased use of robotics is expected in future TKA surgery. However, new high‐quality studies are necessary to confirm the potential benefit of robotic‐assisted TKA from the radiological point of view, but even more to understand the potential translation of these improvements into better clinical outcomes.

## AUTHOR CONTRIBUTIONS

**Christian Nogalo**: Conceptualisation; methodology; data‐curation and synthesis; writing—original draft preparation; writing—review and editing. **Luca Farinelli**: Conceptualisation; methodology; data‐curation and synthesis; writing—original draft preparation; writing—review and editing. **Amit Meena**: Conceptualisation; writing—original draft preparation; writing—review and editing. **Fabrizio di Maria**: Conceptualisation; data‐curation and synthesis; writing—review and editing. **Elisabeth Abermann**: Conceptualisation; writing—original draft preparation; writing—review and editing. **Christian Fink**: Conceptualisation; methodology; data‐curation and synthesis; writing—original draft preparation; writing—review and editing; supervision; all authors interpreted the data, critically reviewed the work, made important contributions to the manuscript with their Title Page suggestions for improvement, approved the published version and agreed to be responsible for all aspects of the work. All authors have read and agreed to the published version of the manuscript.

## CONFLICT OF INTEREST STATEMENT

The authors declare no conflict of interest.

## ETHICS STATEMENT

Performed at the Ethics Committee Innsbruck, Austria (No.: 1149/2023).

## Data Availability

The data sets used and/or analysed during the current study are available from the corresponding author on reasonable request.
